# Essential Oils from Ugandan Medicinal Plants:* In Vitro* Cytotoxicity and Effects on IL-1*β*-Induced Proinflammatory Mediators by Human Gingival Fibroblasts

**DOI:** 10.1155/2016/5357689

**Published:** 2016-10-11

**Authors:** Francis Ocheng, Freddie Bwanga, Elisabeth Almer Boström, Moses Joloba, Anna-Karin Borg-Karlson, Tülay Yucel-Lindberg, Celestino Obua, Anders Gustafsson

**Affiliations:** ^1^Department of Dentistry, School of Health Sciences, College of Health Sciences, Makerere University, P.O. Box 7072, Kampala, Uganda; ^2^Department of Medical Microbiology, School of Biomedical Sciences, College of Health Sciences, Makerere University, P.O. Box 7072, Kampala, Uganda; ^3^Department of Dental Medicine, Unit of Periodontology, Karolinska Institutet, P.O. Box 4064, 141 04 Huddinge, Sweden; ^4^Ecological Chemistry Group, Department of Chemistry, School of Chemical Science and Engineering, Royal Institute of Technology, 100 44 Stockholm, Sweden; ^5^Mbarara University of Science and Technology, P.O. Box 1410, Mbarara, Uganda

## Abstract

The study investigated cytotoxicity of essential oils from four medicinal plants (*Bidens pilosa*,* Ocimum gratissimum*,* Cymbopogon nardus*, and* Zanthoxylum chalybeum*) on human gingival fibroblasts and their effects on proinflammatory mediators' secretion. Cytotoxicity of essential oils was investigated using 3-(4, 5-dimethylthiazol-2-yl)-2,5-diphenyl-tetrazolium bromide assay. Effects of essential oils at subcytotoxicity concentrations on interleukin- (IL-) 6, IL-8, and prostaglandin E_2_ (PGE_2_) secretions by gingival fibroblasts treated with IL-1*β* (300 pg/mL) were evaluated by ELISA and EIA. IC_50_ values of the essential oils ranged from 26 *μ*g/mL to 50 *μ*g/mL. Baseline and IL-1*β*-induced secretion of PGE_2_ was inhibited by treatment with essential oil from* O. gratissimum*. Essential oils from* B. pilosa* and* C. nardus* had synergistic effects with IL-1*β* on PGE_2_ seceretion. In conclusion, the study suggests that essential oil from* O. gratissimum* decreases gingival fibroblasts secretion of PGE_2_, while essential oils from* B. pilosa* and* C. nardus* increase PGE_2_ secretion. Essential oil from* Z. chalybeum* was the most cytotoxic, while oil from* C. nardus* was the least cytotoxic. Although the clinical significance of these findings remains to be determined, it may be suggested that essential oil from* O. gratissimum*, applied at subcytotoxicity concentrations, could reduce the participation of gingival fibroblasts in the gingival inflammation and tissue destruction associated with periodontitis.

## 1. Introduction

Periodontitis is a chronic and destructive inflammatory disease of the tooth-supporting tissues, affecting 11% of the population worldwide [1]. The disease is initiated by accumulation of specific Gram-negative periodontopathic bacteria such as* Porphyromonas gingivalis* and* Aggregatibacter actinomycetemcomitans* in the gingival crevice, which in susceptible individuals cause tissue degrading inflammation that can destroy the periodontal tissues and eventually lead to tooth loss [2]. The continuous secretion of proinflammatory mediators, including interleukin- (IL-) 1*β*, IL-6, IL-8, and tumour necrosis factor-a (TNF-*α*), as well as of prostaglandin E_2_ (PGE_2_), by immune and resident cells in response to bacteria and their products, is a critical determinant of disease progression [3, 4].

Conventional treatment of periodontitis relies on mechanical therapy aimed at minimising or eliminating microbial biofilm [5]. However, not all patients respond well to such treatment and some continue to experience disease progression with attachment loss in spite of good oral hygiene [5]. A new and more efficient treatment option, based on the modulation of the inflammatory response, together with direct control of the microbial biofilm, has been proposed for the management of periodontitis [6]. Consequently, active compounds endowed with both the capacity to modulate host inflammatory response and the control of microbial biofilm are now receiving considerable attention as they may represent potential new therapeutic agents for treating periodontitis [7, 8].

Medicinal plants are rich sources of biologically active compounds and they offer opportunities for innovation in drug discovery [9]. In recent years, studies on the biological activities of extracts from plants have become increasingly important in the search for natural and safe alternative medicines [10], with increased interest particularly in essential oils derived from aromatic medicinal plants [11, 12]. Essential oils are odorous, volatile products of plant secondary metabolism and they are found in leaves, stems, seeds, flowers, or other parts of aromatic plants [11]. For decades, essential oils have been recognised to exhibit notable biological activities, including antioxidant [13, 14] and antimicrobial [15] attributes. Some essential oils, such as that from the plant* Melaleuca alternifolia*, also exhibit anti-inflammatory activities [16, 17].

Previously, we studied the antibacterial effects of essential oils extracted from ten aromatic medicinal plants on a panel of Gram-negative bacteria associated with periodontal disease and Gram-positive bacteria associated with dental caries [18]. Several of the essential oils in that study exhibited strong antibacterial effects, particularly on* P. gingivalis* and* A. actinomycetemcomitans* that are known to be involved in the initiation of periodontitis [18]. We hypothesised that since essential oils contain many components, some of these oils could be endowed with both antibacterial and anti-inflammatory capacities. Furthermore, the cytotoxicity of these oils to the host cells was an important consideration as reports have shown that some plants used as food or in traditional medicine are potentially toxic [19, 20].

Gingival fibroblasts, once activated, release mediators that contribute to inflammatory responses and ultimately tissue destruction in periodontitis [3]. When challenged with IL-1*β* and TNF-*α*, gingival fibroblasts produce prostaglandins E_2_ that stimulate bone resorption [21, 22]. There is also evidence that gingival fibroblasts, challenged with* A. actinomycetemcomitans* from early-onset periodontitis (EOP) patients, are capable of secreting considerable amounts of IL-6 and IL-8* in vitro* [23]. Clinical studies have shown increased levels of IL-6 in gingival tissues of patients with periodontitis compared with healthy controls [24]. IL-6 is considered a key molecule in the promotion of osteoclastogenesis and bone resorption and IL-6 receptor antagonist strongly reduces bone erosion* in vivo* [25]. On the other hand, IL-8 is a potent chemokine that is important in acute inflammation and directs migration of polymorphonuclear (PMN) leukocytes, monocytes, and macrophages to sites of infection [26]. Clinically, increased expression of IL-8 expression has been shown to be localized to sites with higher concentration of PMN cells in gingival tissues from patients with periodontitis [27]. Through their involvement in the production of proinflammatory mediators, gingival fibroblasts therefore act as accessory immune cells and thereby participate in the periodontal tissue destruction.

The present study was undertaken to investigate the cytotoxicity of essential oils from four plants previously showing promising antibacterial effects (namely,* Bidens pilosa*,* Ocimum gratissimum*,* Cymbopogon nardus*, and* Zanthoxylum chalybeum*) and to test their potential effects on the production of proinflammatory mediators (IL-6, IL-8, and prostaglandin E_2_) by human gingival fibroblasts challenged with IL-1*β*.

## 2. Materials and Methods

### 2.1. Plant Essential Oil and Preparation

The essential oils from the following plants were selected for the study:* Bidens pilosa*,* Cymbopogon nardus*,* Zanthoxylum chalybeum*, and* Ocimum gratissimum*. The oils were selected because of the previously described antibacterial effects on the Gram-negative periodontopathic bacteria* A. actinomycetemcomitans* and* P. gingivalis* [18]. The collection of plants and extraction of the essentials oil are detailed elsewhere [18].

### 2.2. Chemicals

Dulbecco's Modified Eagle's Minimum Essential Medium (DMEM), Phosphate Buffer Saline (PBS) (without calcium and magnesium), Fetal Bovine Serum (FBS), trypsin (0.25%), and Penicillin-Streptomycin-Glutamine (50 mg/mL) were purchased from Invitrogen Life Technologies (Paisley, UK); 3-(4, 5-dimethylthiazol-2-yl)-2,5-diphenyl-tetrazolium bromide (MTT) Cell Viability Assay Kit was purchased from Abnova Corporation (Taipei, Taiwan); human IL-6 and IL-8 Duoset enzyme-linked immunosorbent assay (ELISA) kit was purchased from R&D Systems (Minneapolis, MN, USA); prostaglandin E_2_ monoclonal enzyme immunoassay (EIA) kit was obtained from Cayman Chemicals (Ann Arbor, MI, USA).

### 2.3. Gingival Fibroblast Cell Culture

Human gingival fibroblasts cells used in the study were originally established from gingival biopsies obtained from 7 systemically and periodontal healthy donors (aged 7–12) with approval of the Ethical Committee at the Huddinge University Hospital, Stockholm, Sweden (reference number: 377/98). Gingival fibroblasts were established and cultured as described [28]. For the experiments, cells were cultured in DMEM supplemented with 5% FBS and 1% Penicillin-Streptomycin-Glutamine (50 mg/mL), grown as monolayer cultures in corning cell culture flask, 75 cm^2^, (Nunc™, Denmark) and incubated at 37°C and 5% CO_2_. The medium was replaced every 3 to 4 days until about 80% confluence was reached followed by detachment for experimental use. Cells at passages 10–15 were used in all experiments to ensure stability.

### 2.4. Cytotoxicity Assay

Cytotoxicity was assessed by MTT assay where viable cells with active mitochondria reduce the amount of MTT and the value of absorbance obtained by the plate reader is directly proportional to the viability of the cells [29]. Briefly, the human gingival fibroblasts (1 × 10^4^) were seeded in 96-well tissue culture plates (VWR^R^ International, Leven) in 200 *μ*L medium and incubated for 48 hours to allow cell adherence and cells to grow to the exponential phase of growth. The medium was then removed and cells washed twice with serum-free medium and thereafter incubated in serum-free medium with increasing concentrations of the oils solubilised in ethanol. After 24 hours, 100 *μ*L of MTT solution was added and plates were incubated for 4 hours at 37°C. The formazan crystals formed were solubilised with solubilising solution and the absorbance was determined at 620 nm by a microplate spectrophotometer (Labsystem Multiskan MS). The average of the blank control was determined and the amount subtracted from all absorbance values. The concentration at which 50% of the cells were killed (IC_50_) for each essential oil was determined as recommended in the MTT kit. For each essential oil, the experiment was run in triplicate and repeated three times and the average IC_50_ calculated. The lower the IC_50_ value, the more toxic the oil, because less is required to achieve the killing of the cells.

### 2.5. Measurements of IL-6, IL-8, and PGE_2_

For measurement of inflammatory mediators, cells (4 × 10^4^) were seeded in 24-well plates in 300 *μ*L medium and incubated for 24 hours to allow attachment. The medium was removed and cells were rinsed twice with serum-free medium and thereafter stimulated in serum-free media with IL-1*β* (300 pg/mL) alone or in combination with increasing subcytotoxicity concentrations of the oils. Supernatants were collected after 24 hours of incubation and stored at −80°C until analysis. IL-6 and IL-8 levels in the supernatants were measured using the Duoset ELISA kit (R&D Systems Inc., Minneapolis, MN, USA) and PGE_2_ levels determined using PGE_2_ monoclonal enzyme immunoassay (EIA) kit (Cayman Chemicals, Ann Arbor, MI, USA). Both kits were used according to the manufacturer's instructions. Readings were made at 450 nm for IL-6 and IL-8 and at 405 nm for PGE_2_ with microplate spectrophotometer (Labsystem Multiskan MS).

### 2.6. Statistical Analysis

Sigmoidal dose responses and nonlinear regression analyses were undertaken to identify IC_50_ (concentration that causes a reduction by half of the activity of mitochondrial dehydrogenase) values of each essential oil. To evaluate differences in IC_50_ of the essential oils and effects of essential oils on the IL-1*β* induced production of proinflammatory mediators (IL-6, IL-8, and prostaglandin E_2_), one-way ANOVA combined with Tukey's* post hoc* test was used. All statistical analyses were performed using Prism 6 (GraphPad Software, San Diego, CA, USA). Values of *P* < 0.05 were regarded as significant.

## 3. Results

### 3.1. Cytotoxicity Test

We analysed the cytotoxicity (IC_50_ values) of the essential oils from* B. pilosa*,* Cymbopogon nardus*,* Zanthoxylum chalybeum*, and* Ocimum gratissimum* on human gingival fibroblasts presented in Table 1. IC_50_ values differed significantly between the four oils (*P* = 0.0003). Tukey's* post hoc* comparisons test indicated statistically significant differences in mean IC_50_ values of all the oils except that of* Bidens pilosa* and* Ocimum gratissimum*.* Cymbopogon nardus* essential oil was the least cytotoxic. The overall rating of cytotoxicity was thus* Zanthoxylum chalybeum* >* Ocimum gratissimum* and* Bidens pilosa* >* Cymbopogon nardus*.

### 3.2. Effect of Essential Oils on IL-1*β*-Induced IL-6, IL-8, and PGE_2_ in Human Gingival Fibroblasts

**Figure 1:**
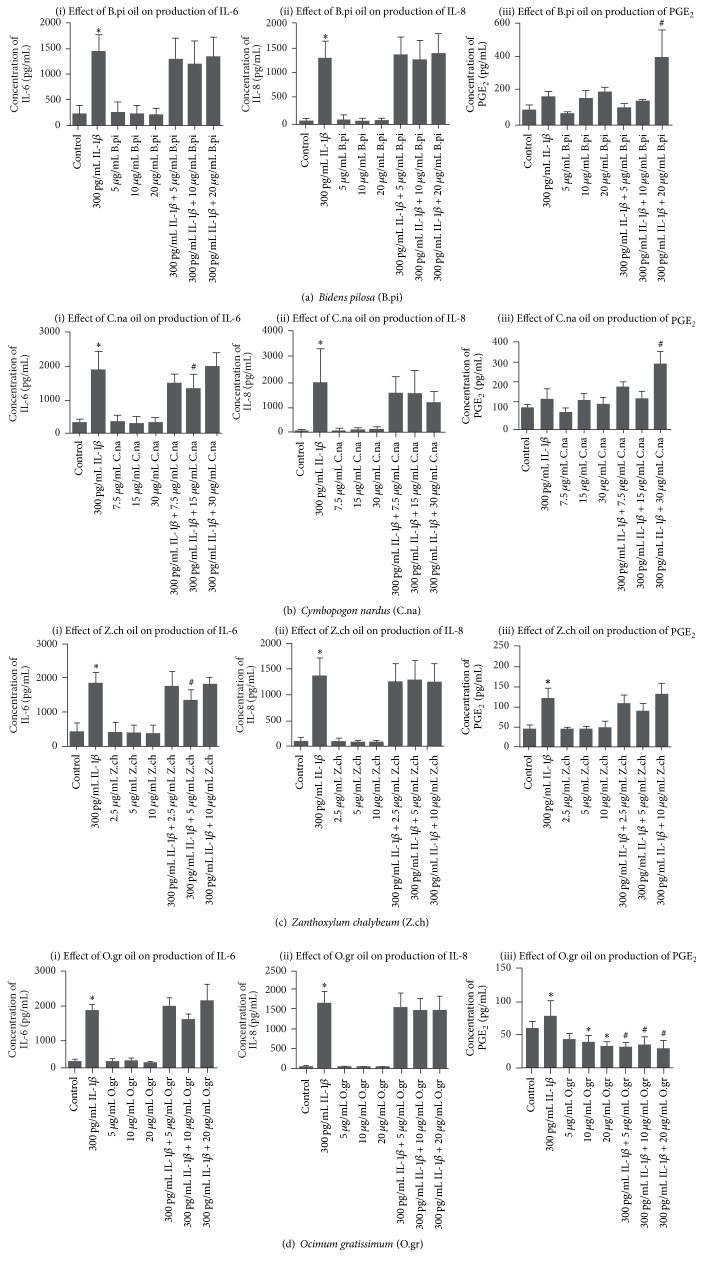
Effects of essential oils from Ugandan medicinal plants on IL-1*β*-induced IL-6, IL-8, and PGE_2_ in human gingival fibroblasts. Human gingival fibroblasts were treated with IL-1*β* alone (300 pg/mL), essential oils at increasing subcytotoxicity concentrations, or combination of IL-1*β* (300 pg/mL) and essential oils. Untreated cells were used as control. IL-6 and IL-8 were assessed by enzyme-linked immunosorbent assay (ELISA). PGE_2_ was assessed by enzyme immunoassay (EIA). The data are the means ± standard deviations of triplicate assays for three independent experiments. Statistical significance was determined using one-way ANOVA and Tukey's* post hoc* test. ^*∗*^ *P* < 0.05 compared with the untreated control; ^#^ *P* < 0.05 compared with cells stimulated with 300 pg/mL IL-*β* alone.

Using human gingival fibroblast cells exposed to IL-1*β* to provoke an inflammatory response, we assessed the effects of essential oils at subcytotoxicity concentrations on IL-6, IL-8, and PGE_2_ secretions. In the absence of IL-1*β* (control), there was baseline secretion of IL-6, IL-8, and PGE_2_ (Figure 1). Cells exposed to IL-1*β* (300 pg/mL) showed, as expected, increased secretion of IL-6, IL-8, and PGE_2_ compared to unexposed cells (Figure 1). Essential oil from* B. pilosa* had no effect on baseline or on IL-1*β*-induced IL-6 and IL-8 secretions (Figure 1(a)(i) and (ii)). However, the oil increased the secretion of PGE_2_ at baseline, but, compared with control, the increase was not statistically significant (Figure 1(a)(iii)). Essential oil from* B. pilosa* had synergistic effects with IL-1*β* on the secertion of PGE_2_ at concentrations of 20 *μ*g/mL (*P* < 0.05) (Figure 1(a)(iii)). Essential oil from* C. nardus* had no effect on baseline secretion of IL-6, IL-8, and PGE_2_ (Figure 1(b)). However, this oil exhibited a statistically significant decrease in IL-1*β*-induced IL-6 secretion specifically at 15 *μ*g/mL concentrations compared to cells exposed to IL-1*β* alone (Figure 1(b)(i)). The oil also exhibited a tendency to decrease IL-1*β*-induced IL-8 secretion with increasing concentrations (7.5, 15, and 30 *μ*g/mL) by 20%, 21%, and 39%, respectively; however, this did not reach statistical significance (Figure 1(b)(ii)). Essential oil from* C. nardus* had synergistic effects with IL-1*β* on the secertion of PGE_2_ specifically at 30 *μ*g/mL concentrations (*P* < 0.05) (Figure 1(b)(iii)). Essential oil from* Z. chalybeum* had no effect on baseline secretion of IL-6, IL-8, and PGE_2_ (Figure 1(c)).* Z. chalybeum* oil, however, exhibited a statistically significant decrease in IL-1*β*-induced IL-6 secretion specifically at 5 *μ*g/mL concentration (*P* < 0.05) (Figure 1(c)(i)). Essential oil from* O. gratissimum* had no effect on baseline secretion of IL-6 and IL-8 (Figure 1(d)(i) and (ii)). The oil, however, decreased baseline secretion of PGE_2_ with increasing concentrations (5, 10, and 20 *μ*g/mL) by 23%, 32%, and 43% respectively, with statistically significant decreases at 10 *μ*g/mL and 20 *μ*g/mL concentrations compared with the control (Figure 1(d)(iii)). The oil also significantly decreased IL-1*β*-induced PGE_2_ secretion by 59–63% (*P* < 0.05), but there was no specific dose-response relationship (Figure 1(d)(iii)).

## 4. Discussion

Due to their long usage in the treatment of diseases in accordance with knowledge accumulated over centuries, medicinal plants are usually presumed to be safe [30]. However, studies have shown some plants used in traditional medicine to be toxic [19, 20]. Thus, it has been recommended that pharmacological studies on medicinal plants should always be followed by toxicological screening on the host cells [31]. Further,* in vitro* cytotoxicity testing of medical/dental materials on host cells is a basic requirement by International Standard Organization (ISO) prior to commencement of other advanced tests [32]. In light of this, we investigated the cytotoxic effects of the essential oils from medicinal plants, namely,* B. pilosa*,* O. gratissimum*,* C. nardus*, and* Z. chalybeum*, that are used traditionally in management of oral diseases in Uganda and have shown marked antibacterial effects on oral pathogens in our previous study [18]. Cytotoxicity testing of the oils was performed on human gingival fibroblast cells because they are the most predominate cells in the oral tissues and thus likely to come in direct contact with the oils, especially in periodontitis cases with ulcerated sulcular mucosa.

In this study, essential oil from* Z. chalybeum* was found to be the most cytotoxic on human gingival fibroblasts with IC_50_ of 26 *μ*g/mL. Other studies have investigated the cytotoxicity of dichloromethane extract from the leaves of* Z. chalybeum* on human leukemia HL-60 cells and reported IC_50_ values of 30 *μ*g/mL [33]. We had earlier found the essential oil of* Z. chalybeum* to contain a high amount of aldehydes compounds geranial (13%) and neral (10%), which are collectively known as citral [18]. The presence of aldehydes in high amounts in foods has been associated with increased cytotoxicity [34]. It is probable that the high cytotoxic effects observed in the* Z. chalybeum* essential oil in this study is due to a high amount of aldehydes.

*C. nardus* essential oil had the least cytotoxic effect on the gingival fibroblasts cells (IC_50_ = 50 *μ*g/mL) of the four essential oils. This oil is composed of mainly sesquiterpenes terpenes with Intermedeol (43.7%) as a major constituent [18]. Other studies found the cytotoxicity of Intermedeol on several cell lines to range from IC_50_ values of 12 *μ*g/mL to 77 *μ*g/mL [35].

*B. pilosa* and* O. gratissimum* essential oils had similar cytotoxic effects on the gingival fibroblast despite having different groups and compounds [18]. Other reports had shown* O. gratissimum* essential oil collected from Benin with thymol (29%) and p-cymene (28%) as major compounds to be less toxic on human fibroblast cell lines (IC_50_ = 166 *μ*g/mL) [36] compared to what is seen in this study (IC_50_ = 36 *μ*g/mL) with eugenol (56.4%) as a major compound. The difference in cytotoxicity could be attributed to differences in chemical compositions of the essential oils which may change according to the habitat (chemotypes), the time point at which the plants are harvested, and the plant growth phase [15].

Essential oils are mixtures of different molecules and each oil may contain between 20 and 70 components, which are usually of low molecular weights and at different concentrations. Most molecules are present in traces, while two to three are often the most representative components, accounting for 20–70% of the whole oils and these may be responsible for determining the biological effects of the essential oil [37] including cytotoxicity. The various active molecules in the essential oils could therefore activate different targets in a cell.

The observed cytotoxicity of the essential oils seen in this study could probably be due to a number of mechanisms including induction of cell death by apoptosis and/or necrosis, cell cycle arrest, and loss of key organelle functions. Some of the effects could be ascribed to the lipophilic and low molecular weight characteristics of the constituents of essential oils. These characteristics allow the oil constituents to cross cell membranes, altering the phospholipid layers, increasing membrane fluidity, and leading to leakage of ions and other cytoplasmic contents. Reduced ATP production, alteration of pH gradient, and loss of mitochondrial potential are just a few of the consequences of disturbed cellular membranes [38].

Chlorhexidine mouth rinse is widely used as an adjunct treatment for periodontitis and is commonly employed as the “gold standard” for evaluating other oral care products. However,* in vitro* studies have shown chlorhexidine to be highly cytotoxic even in low concentration to many cell types, including gingival fibroblasts [39], macrophages [40], and osteoblastic and endothelial cells [41]. Other studies have also shown that topical application of chlorhexidine may result in its penetration through the epithelial barrier, thus triggering tissue damage [42]. The cytotoxicity of chlorhexidine to human gingival fibroblasts has been suggested to be through the inhibition of protein synthesis [43].

Human gingival fibroblasts exposed to IL-1*β* produce inflammatory mediators such as IL-6, IL-8, and PGE_2_ that play important roles in inflammatory responses and tissue degradation [44–46]. IL-6 has the ability to induce osteoclastogenesis [47]. IL-8 acts as a chemoattractant for neutrophils that play a role in the phagocytosis of periodontopathic bacteria [26]. PGE_2_ has several functions, such as vasodilation, the enhancement of vascular permeability, the enhancement of pain, and the induction of osteoclastogenesis, and is believed to play an important role in inflammatory response and alveolar bone resorption in periodontal disease [48]. Therefore, we examined the effects of essential oils from the Ugandan aromatic medicinal plants on IL-1*β*-induced secretions of IL-6, IL-8, and PGE_2_.

An important finding is that essential oil from* O. gratissimum* essential oil significantly decreased both induced PGE_2_ and baseline secretion of PGE_2_ (Figure 1(d)(iii)). The synthesis of PGE_2_ is regulated via 3 groups of enzymes, namely, phospholipase A_2_, cyclooxygenase (COX), and prostaglandin E synthase (PGES). The phospholipase A_2_ enzymes catalyse the conversion of membrane lipids to arachidonic acid, which is further converted to prostaglandin H_2_ (PGH_2_) by the two COX isoforms (COX-1 and COX-2). COX-1 is responsible for the baseline levels of PGE_2_, while COX-2 produces induced PGE_2_. The terminal step from PGH_2_ to PGE_2_ is catalysed by PGES enzymes [48]. Our finding that* O. gratissimum* essential oil decreased both baseline and induced PGE_2_ secretion suggests that the oil acts upstream by inhibition of either the phospholipase A_2_ or the COX enzymes function. We had previously shown that* O. gratissimum* essential oil contains several compounds, the major ones being eugenol (56.4%) and *β*-cubebene (10.9%) [18]. Eugenol had been shown to inhibit arachidonic acid metabolism in platelets via the cyclooxygenase pathway [49]. It is therefore probable that the observed effect of* O. gratissimum* essential oil on both the basal and induced PGE_2_ secretion could partly be due to the major compound eugenol found in this oil. The analgesic properties of eugenol have also been attributed to its ability to inhibit PGE_2_ synthesis [50]. In Uganda, fresh leaves from* O. gratissimum* are traditionally used in the treatment of toothache [51, 52]. Our finding that the oil extracted from the leaves of* O. gratissimum* was able to reduce basal and induced PGE_2_ in gingival fibroblast cells may lend credence to the traditional use of the plant in treatment of toothache. The findings from this study seem to be line with those of Sahouo et al. [53] who found cyclooxygenase activity of sheep seminal vesicles to be inhibited by essential oil from* O. gratissimum* harvested from Cote d'Ivoire [53].

Our data also indicate that essential oils from* B. pilosa* and* C. nardus* had synergistic effects with IL-1*β* on the secretion of PGE_2_, especially at concentrations of 20 *μ*g/mL and 30 *μ*g/mL, respectively (Figures 1(a)(iii) and 1(b)(iii)). There was also a tendency for increased PGE_2_ production at baseline, though this was not statistically significant. These observed effects could be due to the presence of phenolic compounds, aromatic amines, and other antioxidants in these essential oils [18]. These compounds have been reported to have dual effects on cyclooxygenase activity [50, 54, 55]. At low concentrations, they have stimulatory effect on the COX-I- and COX-II-mediated formation of PGE_2_ and other PG products, and, at high concentrations, they are inhibited [54]. The stimulation of cyclooxygenase activity has been attributed in part to its ability to scavenge free radicals, thus protecting the enzymes from self-inactivation [54].

## 5. Conclusion

In conclusion, the study suggests that essential oil from* O. gratissimum* decreases gingival fibroblast secretion of PGE_2_, while essential oils from* B. pilosa* and* C. nardus* increase the PGE_2_ production. The essential oils from the test plants also show varied degree of cytotoxicity to human gingival fibroblasts, with oil from* Z. chalybeum* being the most cytotoxic and oil from* C. nardus* being the least cytotoxic. Although the clinical significance of these findings remains to be determined, it may be suggested that direct application of* O. gratissimum* essential oil at subcytotoxicity concentrations could reduce the participation of gingival fibroblasts in gingival inflammation and tissue destruction associated with periodontitis.

## Figures and Tables

**Table 1:** IC_50_ values of the essential oils on human gingival fibroblasts.

Plant essential oil	Cytotoxicity (IC_50_ values in *µ*g/mL)
*Bidens pilosa* [A]^a^	38 ± 7
*Cymbopogon nardus* [P]	50 ± 4
*Ocimum gratissimum* [L]	36 ± 2
*Zanthoxylum chalybeum* [R]	26 ± 3

^a^Plant family names: [A], Asteraceae; [P], Poaceae; [L], Lamiaceae; [R], Rutaceae.
